# Acute Intake of Plant Stanol Esters Induces Changes in Lipid and Lipoprotein Metabolism-Related Gene Expression in the Liver and Intestines of Mice

**DOI:** 10.1007/s11745-015-4020-1

**Published:** 2015-05-01

**Authors:** Els De Smet, Ronald P. Mensink, Maurice Konings, Gemma Brufau, Albert K. Groen, Rick Havinga, Marleen Schonewille, Anja Kerksiek, Dieter Lütjohann, Jogchum Plat

**Affiliations:** Department of Human Biology, School for Nutrition, Toxicology and Metabolism (NUTRIM), Maastricht University Medical Centre, Maastricht University, PO Box 616, 6200 MD Maastricht, The Netherlands; Department of Pediatrics, University Medical Center Groningen, Groningen, The Netherlands; Institute for Clinical Chemistry and Clinical Pharmacology, University Clinics Bonn, Bonn, Germany

**Keywords:** Cholesterol, Gene expression, Lipids, Lipoproteins, Lymph canulation, Plant stanols, Intestines

## Abstract

The kinetics of plant stanol uptake and routing in 8-week-old C57BL/6J mice were determined after a plant stanol ester gavage. In addition, acute changes in intestinal and hepatic gene expression were investigated. Mice were fed a plant sterol/stanol poor diet from weaning. At the age of 8 weeks, they received an oral gavage consisting of 0.25 mg cholesterol + 50 mg plant stanol esters dissolved in olive oil. Animals were euthanized at different time points. In a second comparable set-up, mesenteric lymph-cannulated versus sham-operated mice received the same oral gavage, which was now deuterium labeled. Intestinal and hepatic sitostanol concentrations increased within 15 min post-gavage. This rapid hepatic appearance was absent in lymph-cannulated mice, suggesting a very fast lymph-mediated uptake. Hepatic mRNA expression of SREBP2 and its target genes rapidly decreased, whereas expression of LXR target genes increased. The intestinal SREBP2 pathway was increased, whereas the expression of LXR target genes hardly changed. The fivefold and sixfold increased expression of intestinal LDLr and PCSK9 is suggestive of TICE activation. We conclude that in C57BL/6J mice plant stanol kinetics are fast, and affect intestinal and hepatic gene expression within 15 min postprandial after lymph-mediated uptake.

**Electronic supplementary material:**

The online version of this article (doi:10.1007/s11745-015-4020-1) contains supplementary material, which is available to authorized users.

## Introduction

In humans, plant sterols and stanols lower intestinal cholesterol absorption, thereby reducing serum low-density lipoprotein cholesterol (LDL-C) concentrations up to 10 % at daily intakes of 2–2.5 g [1]. The exact mechanisms underlying this effect are unknown. Besides competition with cholesterol for incorporation into mixed micelles, which is necessary for intestinal cholesterol absorption [2], other mechanisms extending towards whole body sterol metabolism have been suggested [3]. Within the enterocytes and hepatocytes, there are numerous proteins involved in the transport and metabolism of cholesterol and plant sterols/stanols. For example, overexpression of the human gene encoding ATP-binding cassette transporter G5 and G8 (ABCG5, ABCG8) in the liver and the small intestine of C57BL/6J × SJL F_2_ mice reduced intestinal cholesterol absorption and promoted biliary cholesterol secretion [4]. Although these cholesterol transporter genes are under control of the liver X receptor (LXR), plant sterol and stanol ester feeding increased fecal neutral sterol excretion without changing intestinal LXR expression [5]. Furthermore, intestinal expression of LXR target genes such as Niemann-Pick C1-Like protein 1 (NPC1L1), ABCA1, ABCG5, ABCG8 was not influenced after plant sterol or stanol intake [6]. 

Consumption of plant sterols or stanols might also interfere with intracellular sterol handling, i.e., the incorporation of cholesterol into chylomicrons. In this respect, Liang et al. [7] showed decreased mRNA expression of acetyl-coenzyme A acetyltransferase (ACAT2) and microsomal triglyceride transfer protein (MTTP) after sitosterol feeding in Golden Syrian hamsters. Also, basolateral apolipoprotein B (apoB) secretion by HepG2 and Caco2 cells was decreased after incubation with plant sterols, suggesting a reduced production of lipoproteins by these liver and intestinal cell lines [8]. Although not conclusive, these studies show that plant sterols and stanols affect intestinal and hepatic sterol metabolism in vitro and in various animal models. For the animal data, the absence of consistent effects may relate to the various amounts of plant sterols in the diets, resulting in different tissue and serum concentrations, which may affect pathways underlying the cholesterol-lowering effects of the added plant sterols/stanols. Besides the potentially confounding effect of the background diet, it should also be acknowledged that metabolism in rodents is extremely fast in comparison to man. In this context, Igel and coworkers [9] showed earlier that in mice, intestinal uptake of dietary plant sterols was an extremely fast process, i.e., free plant sterols administered into the stomach were already present in enterocytes 15 min later. In other words, to study in vivo effects of plant sterols and stanols in mice, sampling must occur at short intervals immediately after administration. To gain more insight in the kinetics of plant sterol and stanol distribution, we used C56BL/6J mice, which were fed a plant-sterol-poor and plant-stanol-poor diet from weaning. We were particularly interested to see whether the fast appearance of plant sterols in the enterocytes was also visible in the liver. In addition, the acute effects of plant stanol esters on intestinal and hepatic expression of genes involved in lipid and lipoprotein metabolism were monitored from 0 to 240 min post-gavage. Post-gavage changes in plant sterol and deuterated plant stanol concentrations were examined as well. 

In this study, we show that an acute bolus of dietary deuterium labeled sitostanol provided as sitostanol oleate appeared already after 15 min in the liver. This rapid hepatic appearance was absent in lymph-cannulated mice, suggesting a very fast lymph-mediated uptake, possible via pre-formed available chylomicrons. Also, the expression profiles of genes involved in hepatic and intestinal lipid and lipoprotein metabolism changed rapidly after the gavage. Interestingly, effects on gene expression in liver and intestine were in the opposite direction.

## Materials and Methods

### Study 1: Animals, Diet and Experimental Design

Female C57BL/6J pups (F0) were fed a plant-sterol-poor and plant-stanol-poor diet from weaning, and were used for breeding at the age of 8 weeks. The newborn pups (F1) were fed the same plant-sterol-poor and plant-stanol-poor diet and housed in a light-controlled and temperature-controlled facility with free access to water. At the age of 8 weeks, the mice (10 males/11 females) were given an oral gavage consisting of unesterified cholesterol (0.25 mg) (Sigma, St. Louis, Mo) and plant stanols (50 mg), which were provided as their fatty acid esters, dissolved in 500 μl refined plant-sterol-poor olive oil. Their regular plant-sterol-poor food was removed from the cages 2 h before the start of the gavage to bring the mice into “fasting” condition. The stanol ester mixture used was prepared by RAISIO Nutrition Ltd. and was composed of 70 % sitostanol and 30 % campestanol (Benecol Liquid, Raisio, Finland). Plant stanols were esterified with a fatty acid blend containing 80 % linoleic acid, 15 % oleic acid and 5 % stearic and palmitic acids to produce fat-soluble plant stanol esters. All mice were injected with Temgesic (0.1 mg/kg) [Schering-Plough, Reckitt Benckiser Healthcare (UK) Limited] subcutaneously 30 min before the gavage for pain relief. At seven different time points post-gavage (*T* = 0, 15, 30, 60, 120, 180 and 240 min), mice were anesthetized with isoflurane (1–2 %), directly followed by blood and tissue collection. Blood was taken via heart puncture at the time the mice were sacrificed. There were no repeated blood samples from the same animal, which means that each time point on a curve is composed of data from different animals. The experiment was approved by the Ethical Committee for animal testing of Maastricht University, the Netherlands (project number 2009-129).

### Study 2: Animals, Diet and Experimental Design

For this experiment, 35 male C57BL/6J mice were fed a plant-sterol-poor and plant-stanol-poor diet from weaning, as described for study 1. At the age of 8 weeks, mice were anesthetized and the ductus lymphaticus thoracicus was cannulated proximal from the cisternae magnum via an abdominal approach. The mice in the control group were subjected to a sham operation, leaving the lymph circulation intact. Immediately after surgery, they received the same gavages as used in study 1. The only difference was that we now used d4-plant stanols (50 mg), which were esterified with oleic acid and d6-cholesterol (0.25 mg). A hydrogenation reaction was used to reduce stigmasterol to d4-plant stanols [10]. The esterification of d4-plant stanols was performed by RAISIO Nutrition Ltd, Finland. The plant stanol blend contained 90 % d4-sitostanol, 8 % d4-campestanol and 2 % non-labeled stigmasterol and brassicasterol. Using the deuterated plant stanols and cholesterol enabled us to specifically follow the plant stanols and cholesterol from the gavage into circulation and the tissues over time. The mice remained under anesthesia until they were sacrificed at six different time points post-gavage. This experiment was approved by the Ethical Committee on animal testing of Groningen University, the Netherlands (project number 5356D).

### Sample Collection

In both studies, blood was collected by cardiac puncture into EDTA tubes 2 h after abstaining from food. Plasma was separated from whole blood by centrifugation at 1000×*g* and stored at −80 °C. After sacrificing, the liver was removed, rinsed with phosphate buffered saline (PBS), and stored for mRNA expression analysis and measurement of plant sterol and stanol concentrations. The intestines were removed, carefully rinsed after a midline incision and divided into four segments: the duodenum, jejunum, ileum and colon. Next, each segment was further divided into smaller parts. The first part of the three small intestinal segments was used for mRNA analysis. These samples were immediately frozen in liquid nitrogen. In contrast, the second part of the intestinal segments, which was used to determine the sterol and stanol concentrations, was scraped before freezing in order to obtain an enterocyte-rich sample. All samples were stored at −80 °C.

### Serum and Tissue Concentration of Sterols and Stanols

Hepatic, intestinal and plasma plant sterol (sitosterol and campesterol), plant stanol (sitostanol and campestanol), cholestanol and cholesterol precursor (lathosterol and desmosterol) concentrations were analyzed by gas–liquid chromatography–mass spectrometry (GC–MS), as described previously [11]. D4-plant stanols and d6-cholesterol were measured as described by Lütjohann et al. [9] and Sudhop et al. [12]. All samples from the same animal were always analyzed in the same run.

### RNA Preparation and Real-Time RT-PCR

Total RNA was isolated from the livers, the duodenum, the jejunum and the ileum. After grinding, the lysate was homogenized in RLT buffer. RNA purification was conducted using the RNeasy mini kit (Qiagen, The Netherlands). Reverse transcription was performed with 350 ng total RNA as described [13]. To 2 μl cDNA, 1 μl primer of the gene of interest and 1 μl primer of the household gene were added (Table 1). The PCR mixture also consisted of 6 μl water and 10 μl mastermix (Applied Biosystems). The cDNA was amplified for 40 cycles. The probes from the genes of interest were FAM labeled at the 5′ end. All data was normalized to hypoxanthine phosphoribosyltransferase 1 (HPRT1) (VIC labeled/MGB Probe, Primer Limited; Gibco, Life Technologies). Thus, the expression of the gene of interest and the household gene, i.e., vic-labeled HPRT1, was measured in duplicate in the same run. Next, the average cycle threshold (C_*t*_) was calculated for the gene of interest and for the household gene. Based on the difference between both C_*t*_ values, the comparative was calculated. The comparative of the mice sacrificed at time point 0 was set at 1. The comparative of all the other time points was normalized to the control comparative of time point 0.

**Table 1:** Genes of interest and their specific assay on demand

Gene	Specific assay (applied biosystems, life technologies)
ABCA1	Mm00442646_m1
ABCG5	Mm00446241_m1
ABCG8	Mm00445970_m1
ACAT2	Mm00782408_s1
ApoB	Mm01545156_m1
HMG-CoA reductase	Mm01282499_m1
HPRT1	Mm00446968_m1
LXRα	Mm00443451_m1
MTTP	Mm00435015_m1
NPC1L1	Mm01191972_m1
PCSK9	Mm01263610_m1
SREBP2	Mm01306292_m1

## Results

### Intestinal Cholesterol and Plant Stanol Concentrations

**Fig. 1:**
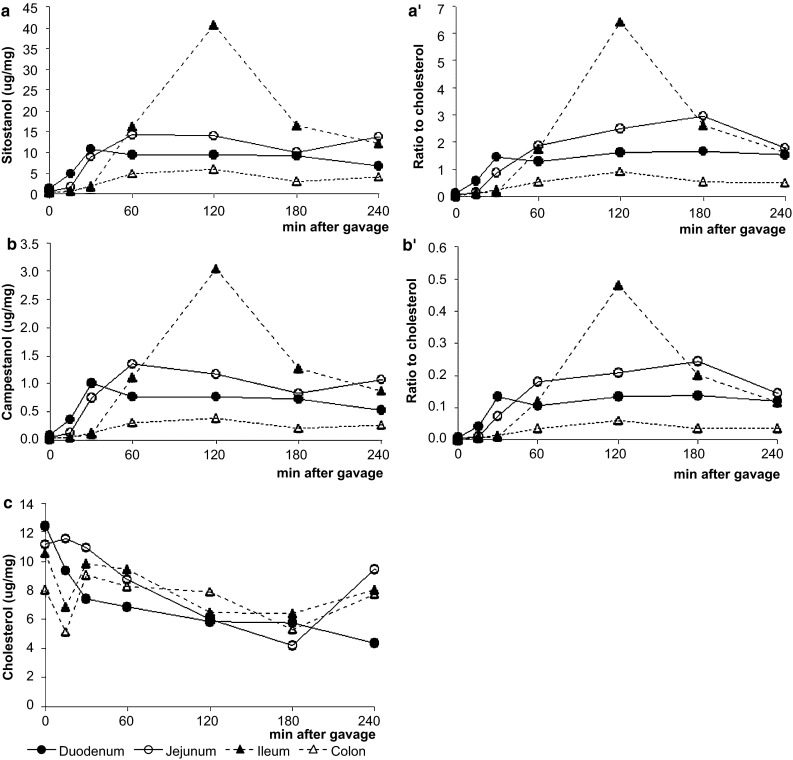
Study 1: time kinetics of sitostanol (**a**, **a′**), campestanol (**b**, **b′**) and cholesterol (**c**) levels in the intestinal tissue at different time points expressed as absolute value and as a ratio to cholesterol. Values are expressed as means (*n* = 2 or 3 each)

**Fig. 2:**
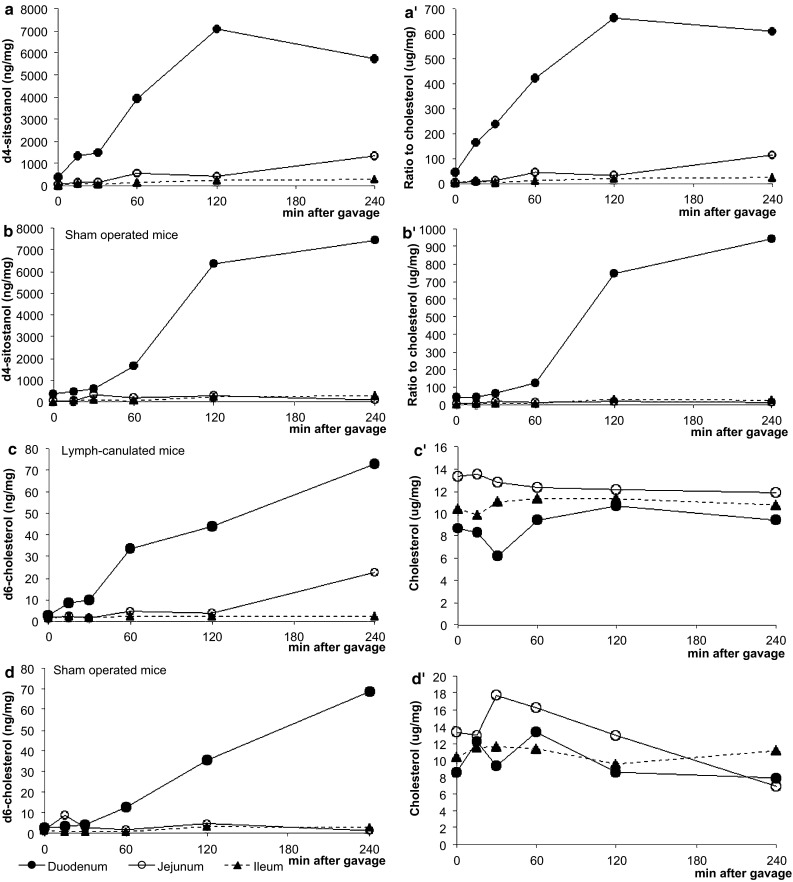
Study 2: time kinetics of d4-sitostanol levels in intestinal tissue of lymph-cannulated mice (**a**, **a′**) and of sham operated mice (**b**, **b′**) at different time points. Results are expressed as absolute concentration and standardized for cholesterol. Each time point represents the mean of two or three animals. Time kinetics of d6-cholesterol (**c**) and cholesterol (**c′**) concentrations of lymph-cannulated mice and of sham-operated mice (**d**, **d′**) are shown in *panel* **c** and **c′**, and **d** and **d′**, respectively

During the 240-min post-gavage period in study 1, there was a clear response in transit time of plant stanols within the scraped enterocytes from proximal to distal along the gastrointestinal tract. As expected, the increase in sitostanol became apparent first in the duodenum, followed by the jejunum, ileum, and finally the colon. Results for absolute (μg/mg wet tissue; Figs. 1, 2a) as well as cholesterol-standardized (Figs. 1, 2a′) levels were comparable. The same patterns were observed for campestanol, but were less pronounced, which could be explained by the composition of the gavage (Fig. 1b, b′). There was a remarkably strong increase in both sitostanol as well as campestanol concentrations in the ileum after 2 h. As expected, the uptake at the apical side between the lymph-cannulated and the sham-operated mice (supporting information in Fig. 1a and b) was comparable. Also, the pattern of d4-sitostanol/d6-cholesterol was comparable to that of d4-campestanol/d6-cholesterol, which was again less pronounced. Although the gavage also contained a small amount of cholesterol, the total cellular cholesterol concentrations in the scraped enterocytes decreased slightly over time (Fig. 1c). However, concentrations of the gavage-derived d6-cholesterol within enterocytes increased over time (study 2; Fig. 2c, d).

### Intestinal Expression Profile of Genes Involved in Sterol Metabolism

**Fig. 3:**
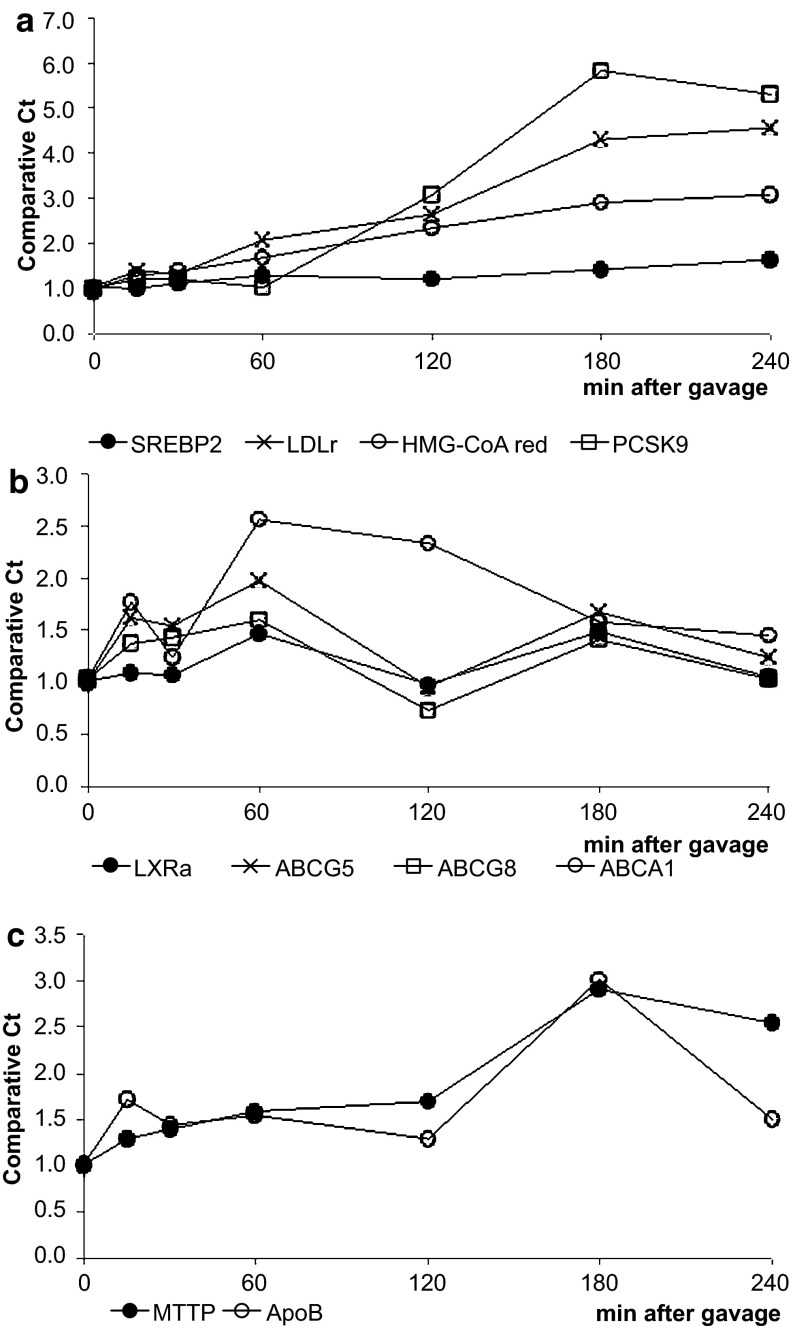
Study 1: changes in the expression profile of genes involved in the sterol metabolism in the duodenum. Values are expressed as means (*n* = 2 or 3 each)

**Fig. 4:**
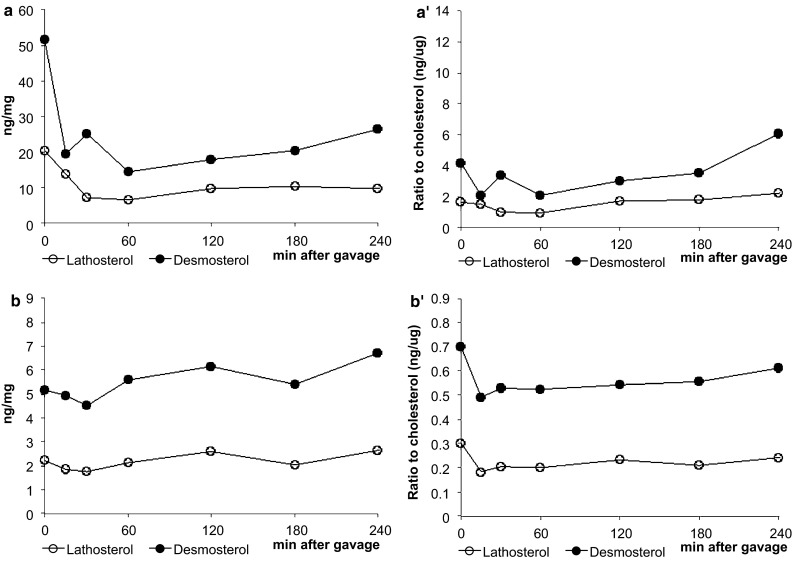
Study 1: time kinetics of lathosterol and desmosterol levels in the duodenum (**a**, **a′**) and in the liver (**b**, **b′**) at different time points. Results are expressed as absolute concentration and standardized for cholesterol. Each time point represents the mean of two or three animals

The intestinal mRNA expression of sterol regulatory element binding protein 2 (SREBP2) and its target genes, 3-hydroxy-3-methyl-glutaryl-CoA reductase (HMG-CoA reductase), the low-density lipoprotein receptor (LDLr) and proprotein convertase subtilisin/kexin type 9 (PCSK9), were clearly upregulated (Fig. 3a) during the post-gavage period. However, the increase in mRNA expression of HMG-CoA reductase did not result in an increase in lathosterol and desmosterol concentrations (Fig. 4a). There was no clear consistent change in the duodenal expression profiles of LXRα and its target genes ABCG5, ABCG8 and ABCA1 throughout the post-gavage period (Fig. 3b). Finally, both intestinal apoB and MTTP expression slightly, but gradually, increased over time for 3 h following the gavage (Fig. 3c).

### Serum and Hepatic Cholesterol and Plant Stanol Concentrations Over Time

**Fig. 5:**
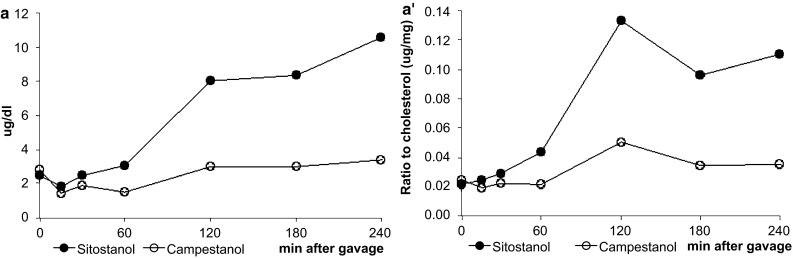
Study 1: time kinetics of sitostanol and campestanol levels in the serum at different time points expressed as an absolute value and as a ratio to cholesterol. Values are expressed as means (*n* = 2 or 3 each)

**Fig. 6:**
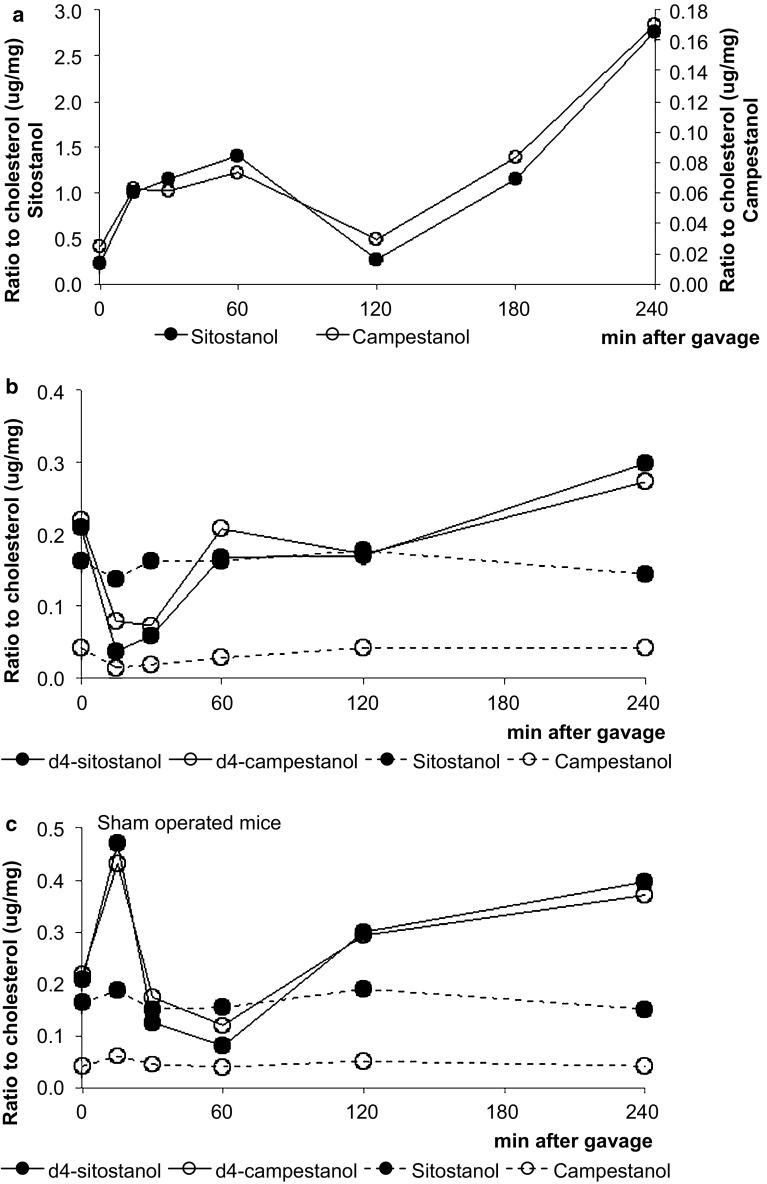
Study 1: time kinetics of sitostanol and campestanol levels in the liver at different time points expressed as μg/mg cholesterol (**a**). Study 2: time kinetics of D4-sitostanol, sitostanol, D4-campestanol and campestanol levels in the liver of lymph-cannulated mice (**b**) and in the liver of sham operated mice (**c**) at different time points. Values are expressed as means (*n* = 2 or 3 each)

In study 1, serum sitostanol and campestanol concentrations clearly increased during the hours following the oral gavage. This increase in serum concentrations started from 30 to 60 min post-gavage (Fig. 5) and continued over the following hours. Surprisingly, hepatic sitostanol concentrations were already increased after 15 min (Fig. 6a), i.e., even before the increase in serum sitostanol concentrations became evident. After this first rapid appearance, sitostanol concentrations decreased and increased again after 120 min. Hepatic campestanol concentrations followed the same pattern, but like for the enterocytes, changes were less pronounced.

The intriguing question is now via which route the plant stanols reached the liver after just 15 min. Interestingly, this very rapid hepatic appearance of the plant stanols was absent in the lymph-cannulated mice in study 2 (Fig. 6b), whereas it was again clearly visible in the sham-operated mice (Fig. 6c). From the data in study 2, it is evident that the hepatic plant stanols were derived from the gavage, since the gavage contained d4-plant stanols that could be detected in the liver. All changes in serum and hepatic plant stanol concentrations occurred without changing hepatic and serum cholesterol concentrations (supporting information, Fig. 2a and b). Finally, we were not able to detect d6-cholesterol in the liver in the post-gavage period.

### Hepatic Expression Profile of Genes Involved in Sterol Metabolism

**Fig. 7:**
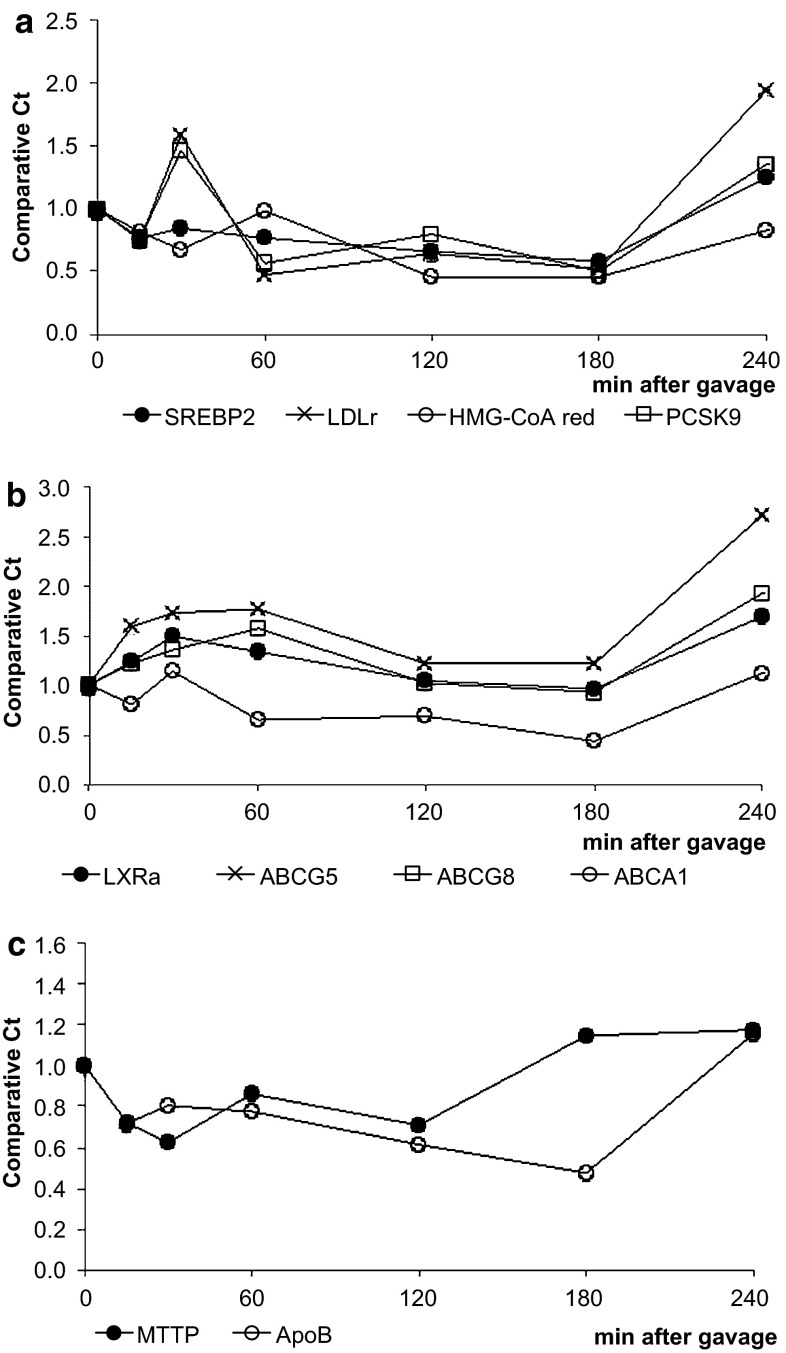
Study 1: changes in the hepatic expression profile of genes involved in sterol metabolism. Values are expressed as means (*n* = 2 or 3 each)

In the liver, mRNA expression levels of SREBP2 and its target genes HMG-CoA reductase, the LDLr and PCSK9 were all very rapidly down-regulated, after only 15 min (Fig. 7a). The hepatic concentration of lathosterol and desmosterol remained practically stable (Fig. 4b). In contrast to the downregulation of the SREBP2 pathway, hepatic expression profiles of LXRα and its target genes ABCG5 and ABCG8 were already upregulated again, starting after 15 min (Fig. 7b). Finally, mRNA levels of apoB and MTTP were immediately downregulated post-gavage (Fig. 7c).

### Serum, Hepatic and Intestinal Cholestanol Concentrations Over Time

**Fig. 8:**
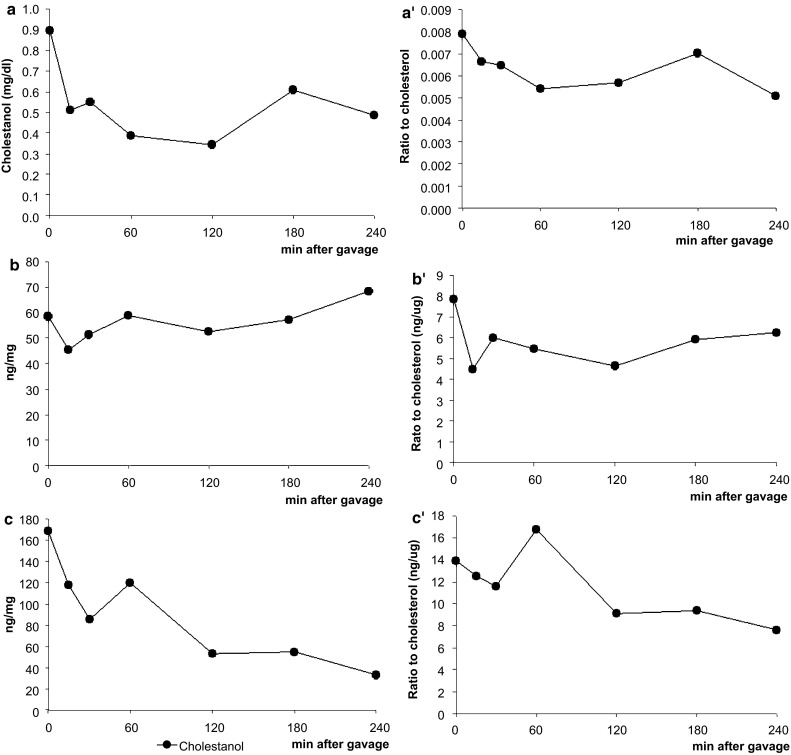
Study 1: time kinetics of cholesteranol levels in the serum (**a**, **a′**), the liver (**b**, **b′**) and the duodenum (**c**, **c′**) at different time points, expressed as absolute value (**a**–**c**) and as a ratio to cholesterol (**a′**–**c′**). Values are expressed as means (*n* = 2 or 3 each)

The ratio of cholestanol to cholesterol can be used to estimate intestinal cholesterol absorption, because this marker is independent of the amount of plant sterols in the diet. However, the use of cholestanol as a marker for cardiovascular disease (CVD) risk is still controversial [14, 15]. We observed that the cholestanol curve in the serum (Fig. 8a) and in the duodenum (Fig. 8c) followed more or less the same pattern as the cholesterol curve.

## Discussion

In this study, we show that in C57BL/6J wild-type mice, hepatic sitostanol and campestanol concentrations increased after 15 min following an oral gavage with (deuterated) plant stanol esters and cholesterol. This rapid hepatic appearance was absent in lymph-cannulated mice. Therefore, our data suggest that plant stanols can be taken up via a very fast lymph-mediated route, possibly via available preformed intestinal chylomicrons. Interestingly, changes in serum plant stanols lagged behind and became evident after 30–60 min. In the intestine, the SREBP2 pathway was activated, whereas expression of LXRα and its target genes remained practically unchanged during the post-gavage period. The increase in intestinal LDLr and PCSK9 expression was especially pronounced. Surprisingly, changes in hepatic gene expression were opposite to those in the intestine. It should be noticed that these acute effects after a one-time single dose of plant stanol esters are different from those observed after longer-term intake of plant stanol esters [16]. Moreover, in humans we have shown earlier an increased LDLr expression in peripheral blood mononuclear cells—which correlate positively to that in the liver [17]—after 8 weeksof plant stanol ester consumption [18]. Questions that are not answered by our studies are: (1) how do plant stanols reach the liver so rapidly after intake in a lymph-dependent way without a clear increase in serum concentrations; (2) do the plant stanols reach the liver in free or esterified form; and (3) does the acute change in hepatic plant stanol concentrations affect liver function?

As expected, there was a clear response in transit time of plant stanols within the enterocytes from proximal to distal along the gastrointestinal tract. This pattern was highly consistent and might relate to the fact that we fed the mice plant-sterol-poor diets from weaning to start the oral gavage with very low background plant sterol concentrations in serum as well as in tissues. In Table 2, we compared serum and tissue plant sterol concentrations in three different studies using diets containing different plant sterol contents. It is evident that lower plant sterol contents result in lower concentrations in various tissues. Therefore, it could be argued that differences in dietary plant sterol content might be a main reason for the large inconsistency between these studies. As shown in Fig. 1, in our hands, when using the plant-sterol-poor diets preceding the experimental day, 15 min after the oral gavage, the sitostanol concentration started to increase in the proximal parts of the small intestine. This is in agreement with observations from Igel and colleagues [9], who also detected deuterated sterols and stanols in the small intestinal wall 15 min after administration via a stomach tube, indicating that the uptake in the enterocytes is a rapid process in mice. Unexpectedly, plant stanol concentrations strongly increased in the ileum 2-h post-gavage.

**Table 2:** Comparison of serum and liver concentration of plant sterols after administration of diets different in plant sterol content

	Standard chow [33]	Unpublished data^a^	Our study (F1)
Diet			
Campesterol (ng/ml)	199	63.8	9.85
Sitosterol (ng/ml)	582	219	54.5
Serum			
Campesterol (mg/dl)	3.60 ± 0.76	1.22 ± 0.17	0.70 ± 0.26
Sitosterol (mg/dl)	1.00 ± 0.20	0.52 ± 0.07	0.35 ± 0.14
Liver			
Campesterol (ng/mg)	247 ± 40	102 ± 17	72 ± 15
Sitosterol (ng/mg)	52.8 ± 8.0	28 ± 4.1	18 ± 3.3

^a^Unpublished data. C57BL/6J mice were fed a plant-sterol-poor chow, a plant-sterol-enriched or a plant-stanol-enriched diet for 1 week. After sacrificing, blood and tissues were collected and analyzed. Mice in this study received a plant-sterol-poor chow

This can be explained by the fact that the proximal part of the small intestine is the major site of chylomicron formation and secretion, resulting in fast disappearance of plant stanols within the enterocyte of the duodenum and jejunum. In the more distal parts, chylomicron synthesis is less, resulting in a transient accumulation of plant stanols [19] that fades away when ABCG5/ABCG8 activity increases [20].

In this study, we have shown that within the same time frame, plant stanol concentrations were also strongly elevated in the liver, suggesting that plant stanol uptake and distribution is even faster than indicated by Igel et al. [9]. This extremely rapid hepatic appearance of sitostanol was unexpected, since it suggests that it only takes 15 min for the plant stanol esters to be digested and absorbed into the enterocytes, incorporated into chylomicrons, secreted into the lymph, and removed by the liver after entering the circulation. We therefore propose that this very fast lymph-mediated uptake should be facilitated via available preformed intestinal chylomicrons. Coppack et al. [21] described earlier the possibility of releasing such chylomicrons following ingestion of carbohydrate as well as mixed meals. Surprisingly, there was no clear change in serum plant stanol or cholesterol concentrations preceding the hepatic appearance at this early time point. It cannot be excluded that the enrichment of plant stanols in serum was too low to be detected at this stage due to a strong dilution. If true, this dilution must have been lower in the liver, making detection possible. The second, larger increase in hepatic plant stanol concentrations after 120 min might be explained by the uptake of chylomicron remnants by the liver. Theoretically, it is possible to explain this early increase in hepatic concentrations by postulating that plant stanols not only reach the liver via the “normal” chylomicron route, i.e., via secretion into lymph, but also through the portal vein, independent of chylomicron incorporation. Therefore, a second study was performed to specifically address the route of entrance into the liver. In that study, we found that the rapid appearance of d4-plant stanols in the liver was absent in the lymph-cannulated mice. However, the uptake into the enterocytes was comparable between the lymph-cannulated and the sham-operated mice. Therefore, we must conclude that the rapid appearance of plant stanol esters in the liver is lymph-dependent. Interestingly, we were not able to detect d6-cholesterol in the liver within this short time frame, suggesting that the hepatic appearance was specific for plant stanols. However, it could also be possible that the detection limit for d6-cholesterol was too low due to a strong dilution. In line with the observed reduced post-gavage cholesterol content of the scraped enterocytes in the duodenum, the expression of SREBP2 [22] and its target genes increased. Remarkably, the hepatic SREBP2 pathway was downregulated. Whether this will affect metabolism is not known, as changes in mRNA expression are not always translated into changes in protein expression and activity. Therefore, we can only speculate why gene expression in these two tissues differed. In the intestine, intracellular cholesterol concentrations post-gavage decreased, which might have activated the SREBP2 pathway. In the liver, the expression of LXR target genes ABCG5 and ABCG8, both involved in sterol efflux, was increased. The question remains whether the increased hepatic LXR expression can be explained by an effect of changes in intracellular cholesterol concentrations, or maybe via a direct effect of sitostanol. In this respect, both intestine and liver showed a rapid increase in sitostanol concentrations. Therefore, it is not likely that sitostanol itself will be responsible for the changes in gene expression. 

There might, however, be an alternative explanation. Spann et al. [23] recently showed that desmosterol was an important regulator in LXR activation in macrophages. We observed that desmosterol concentrations in the intestines were severely reduced by 15 min post-gavage, whereas those in the liver remained stable. In this respect, the large difference in absolute desmosterol concentrations between liver and intestine was remarkable. Therefore, it could be speculated that the differences in desmosterol concentrations might have influenced tissue-specific LXR expression. However, It should be noticed that there was a time delay of several hours between the decrease in intestinal desmosterol concentrations and the changes in the expression profile of LXR. Finally, we found a decrease in the hepatic expression profile of MTTP and apoB, suggesting a reduced hepatic lipoprotein production, which is in line with earlier cell [8] and human studies [24].

The fivefold increased intestinal LDLr expression is suggestive for an enhanced clearance of cholesterol via the enterocytes. Le May et al. [25] showed that LDL provides cholesterol to the intestine for transintestinal cholesterol excretion (TICE), which contributes up to 33 % of total fecal sterol loss in mice. Recently, Davidson and colleagues [26] also demonstrated a role for LDL particles in the delivery of cholesterol for TICE. Moreover, Brufau et al. [27] earlier showed an increase in TICE activity after plant sterol intake. Therefore, it may be possible that the increased intestinal LDLr expression, observed in our study, contributes to plant-stanol-induced TICE activation. Recently, a role for not only intestinal LDLr expression, but also for PCSK9, was suggested in TICE [25]. Interestingly, PCSK9 was the strongest upregulated gene we evaluated in our study. Preclinical [28] as well as clinical studies [29] indicate that blocking PCSK9, thereby increasing the number of available LDL receptors, is an attractive route to lower LDL-C levels. More research is, however, needed to unravel the role of PCSK9 after consumption of plant stanols, especially in humans. Finally, if activation of TICE by plant stanols, thereby increasing the clearance of cholesterol through intestinal LDLr upregulation and neutral sterol secretion into the intestinal lumen, contributes to the mechanism behind the LDL-C reductions, this may also explain why no clear reductions on chylomicron formation in humans are observed after plant stanol ester consumption [30, 31]. In other words, it is possible that increased secretion and reduced intestinal cholesterol absorption explain the cholesterol-lowering activity of plant stanols. However, the suggested mechanism via TICE needs to be further elucidated. In this respect, it should be mentioned that despite the strong increase in intestinal LDLr expression, the cholesterol concentrations in the serum and within the enterocyte did not change. Also, how these results compare to the human situation warrants further study.

The ratio of cholestanol to cholesterol has been used to estimate intestinal cholesterol absorption. Though controversial, increased levels of this marker have also been associated with an increased risk for CVD [14, 15]. One advantage of this marker—in contrast to, for example, the campesterol/cholesterol ratio as marker for cholesterol absorption—is that it can be used when plant sterol consumption is increased, as cholestanol cannot be formed from plant sterols. However, there is a clear lack of knowledge regarding cholestanol metabolism. Serum cholestanol originates from dietary intake, as well as from bacterial formation [32]. In our study, serum cholestanol concentrations largely followed over time the same pattern as observed for cholesterol, which supports the finding of Miettinen et al. [32] that serum cholesterol and cholestanol correlate positively in humans. However, from our data, it cannot be concluded whether or not cholestanol is a valid marker to measure intestinal cholesterol absorption, as this latter parameter was not measured in our study.

In summary, we have demonstrated that orally applied plant stanols had a fast appearance within the enterocytes, and in addition were rapidly taken up into the liver. This rapid hepatic appearance could not be observed in the lymph-cannulated mice, suggesting a lymph-dependent route of entrance. Post-gavage changes in hepatic gene expression patterns of genes involved in sterol metabolism were opposite to those of the intestines, indicating that acute effects of plant stanols are tissue specific. Finally, in the acute condition, intestinal LDLr and PCSK9 expression were strongly increased, for which we do not yet oversee the role in the changes in cholesterol metabolism towards longer-term interventions, but this certainly demands further attention in future studies.

## Electronic supplementary material

Supplementary material 1 (DOCX 44 kb)

Supplementary material 2 (PDF 84 kb)

Supplementary material 3 (PDF 74 kb)

## Abbreviations

ABC: ATP-binding cassette transporter
ABCA1: ATP-binding cassette transporter A1
ABCG5: ATP-binding cassette transporter G5
ABCG8: ATP-binding cassette transporter G8
ACAT2: Acetyl-coenzyme A acetyltransferase
apoB: Apolipoprotein B
ATP: Adenosine triphosphate
Caco2: Colon carcinoma cell line
CVD: Cardiovascular disease
HepG2: Human hepatocellular liver carcinoma cell line
HMG-CoA reductase: 3-Hydroxy-3-methyl-glutaryl-CoA reductase
HPRT1: Hypoxanthine-guanine phosphoribosyltransferase
LDL-C: Low density lipoprotein cholesterol
LDLr: Low density lipoprotein receptor
LXR: Liver X receptor
MTTP: Microsomal triglyceride transfer protein
NPC1L1: Niemann-pick C1-like 1
PCSK9: Proprotein convertase subtilisin/kexin type 9
SREBP2: Sterol regulatory element binding protein 2
TAG: Triacylglycerol
TICE: Transintestinal cholesterol excretion
