# Supplementary material for: Acute Intake of Plant Stanol Esters Induces Changes in Lipid and Lipoprotein Metabolism-Related Gene Expression in the Liver and Intestines of Mice
Source: Lipids. 2015 May 1;50(6):529–41. doi: 10.1007/s11745-015-4020-1 (PMC4445258; doi:10.1007/s11745-015-4020-1)
Supplement: Supplementary file 1 — Supplementary material 1 (DOCX 44 kb) [file 11745_2015_4020_MOESM1_ESM.docx]

Supplemental table 1. Genes of interest and their specific assay on demand

| Gene | Specific assay (Applied Biosystems, Life Technologies) |
| --- | --- |
| ABCA1 | Mm00442646_m1 |
| ABCG5 | Mm00446241_m1 |
| ABCG8 | Mm00445970_m1 |
| ACAT2 | Mm00782408_s1 |
| ApoB | Mm01545156_m1 |
| HMG-CoA reductase | Mm01282499_m1 |
| HPRT1 | Mm00446968_m1 |
| LXRα | Mm00443451_m1 |
| MTTP | Mm00435015_m1 |
| NPC1L1 | Mm01191972_m1 |
| PCSK9 | Mm01263610_m1 |
| SREBP2 | Mm01306292_m1 |
