# Supplementary material for: Acute Intake of Plant Stanol Esters Induces Changes in Lipid and Lipoprotein Metabolism-Related Gene Expression in the Liver and Intestines of Mice
Source: Lipids. 2015 May 1;50(6):529–41. doi: 10.1007/s11745-015-4020-1 (PMC4445258; doi:10.1007/s11745-015-4020-1)
Supplement: Supplementary file 2 — Supplementary material 2 (PDF 84 kb) [file 11745_2015_4020_MOESM2_ESM.pdf]

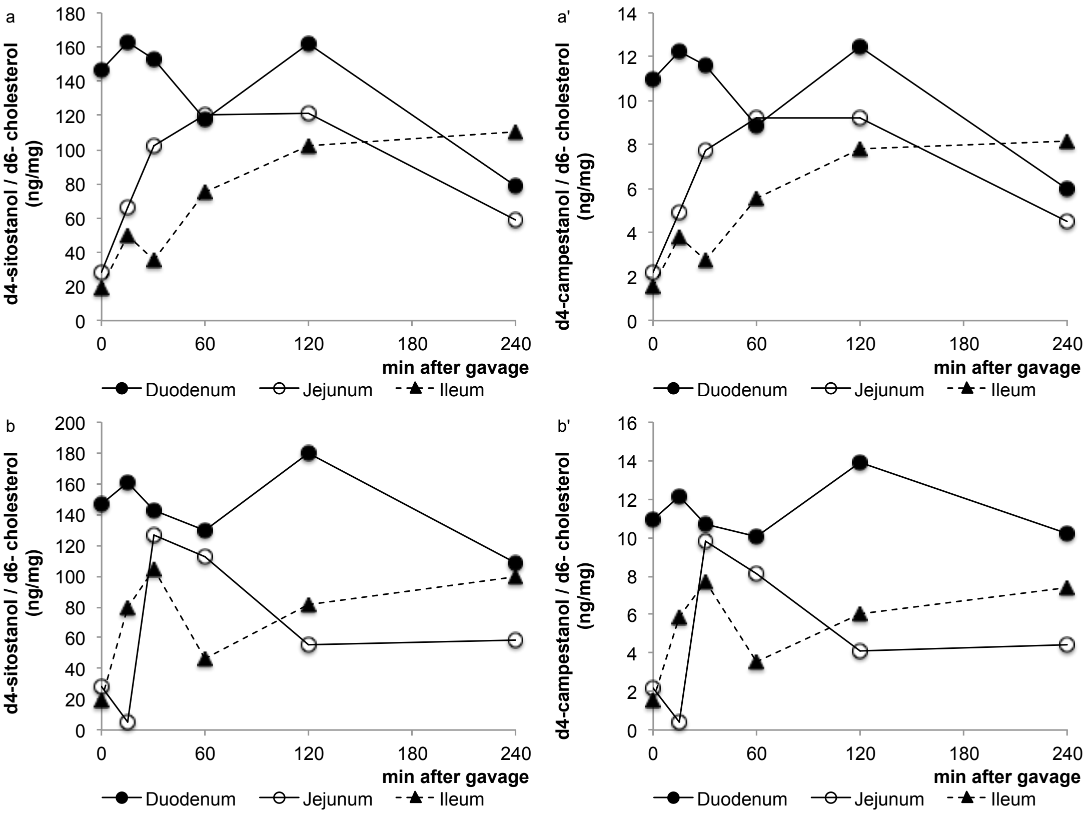

Supplemental figure 1. Study II: Time kinetics of d4-sitostanol/d6-cholesterol and d4-campestanol/d6-cholesterol levels in the intestinal tissue of lymph-canulated (a+a') and of sham operated mice (b+b') at different time points. Each time point represent the mean of 2 or 3 animals.
