# Supplementary material for: Acute Intake of Plant Stanol Esters Induces Changes in Lipid and Lipoprotein Metabolism-Related Gene Expression in the Liver and Intestines of Mice
Source: Lipids. 2015 May 1;50(6):529–41. doi: 10.1007/s11745-015-4020-1 (PMC4445258; doi:10.1007/s11745-015-4020-1)
Supplement: Supplementary file 3 — Supplementary material 3 (PDF 74 kb) [file 11745_2015_4020_MOESM3_ESM.pdf]

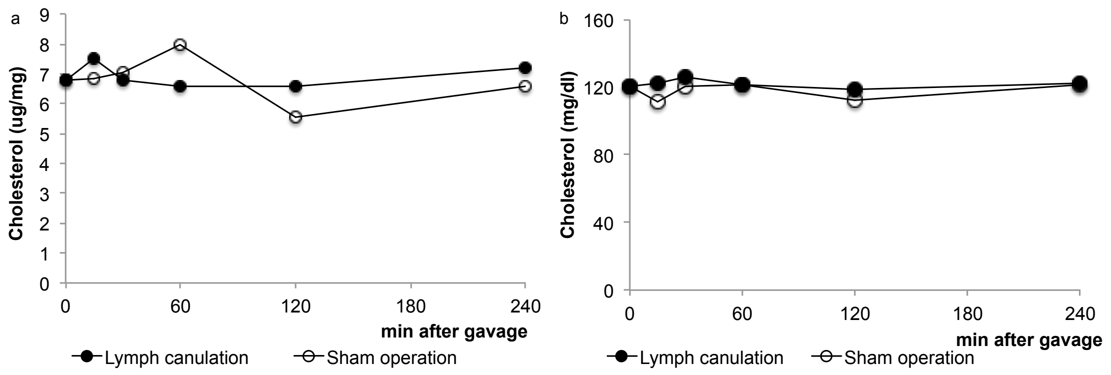

Supplemental figure 2. Study II: Time kinetics of cholesterol concentration in the liver (a) and in the serum (b) at different time points post-gavage. Results are expressed as an absolute concentration. Each time point represent the mean of 2 or 3 animals. The cholesterol concentration was measured in the liver and serum of lymph-canulated mice (LC, closed circles) and sham operated mice (open circles).
